# Death from Chloroform

**Published:** 1872-11

**Authors:** 


### Death from Chloroform.

Mr. W. F. Morgan, M. R. C. S., writes to the *British Medical Journal*, June 1, about chloroform: I fully believe that failure of the heart is the great source of danger in its administration; and that electricity, cautiously employed, is the most prompt and certain remedial measure. I shall not soon forget a striking example in the case of an old man admitted with strangulated hernia. The taxis under ordinary circumstances having failed, he was put under chloroform for another trial previously to operation. The hernia was now readily reduced, but immediately he became deadly pale, his pulse stopped, and he seemed in *articulo mortis.* Instant recourse was had to the electro-magnetic battery, and almost as instantly the pulse returned, and the man was safe.

In my experience, whenever dangerous symptoms have arisen, the heart has been the organ effected; and that is, I believe, the result to be watched and provided for when chloroform is administered. I can readily imagine that the lungs may suffer and asphyxia be produced by complicated apparatus and falling valves, mechanically suffocating the patient. Nothing can be more simple, and in my judgment more safe and effectual, than the plan adopted at the Bristol Royal Infirmary. A hollow sponge, conical in shape, with a hole at the apex, is held loosely over the mouth and nostrils, the chloroform being sprinkled on the interior, with or without the intervention of a bit of lint, the admission of air being regulated by a thumb on the hole at the apex. In the two deaths from chloroform which have occurred in the infirmary, only a very moderate quantity was used, as if the unfortunate result was due to some idiosyncracy.

### Aldini Prize on Electricity.

The Academy of Sciences in Bologna has announced that a prize of 1200 *lire* (£48) will be awarded to the author of the best scientific experimental essay on galvanism or dynamic electricity. Essays intended for the competition must be sent in between July 1, 1872, and July 30, 1874, and must be written in Italian, Latin, or French. They may be either written or printed; but, in the latter case, must not have been published previous to the two years above mentioned. Each essay is to bear a motto, and to be accompanied with an envelope, stating the name of the author. They must be addressed to the Perpetual Secretary of the Academy of Sciences of the Bologna Institution.

